# Differences in Performance and Conductivity Persistence of New Reduced Graphene Oxide Air Filter Materials before and after Eliminating Static Electricity

**DOI:** 10.3390/ma16227146

**Published:** 2023-11-13

**Authors:** Yun Gao, Huixin Shi, Xin Zhang, Jingyao Ma, Tao Yu

**Affiliations:** 1 School of Resources Engineering, Xi’an University of Architecture and Technology, Xi’an 710055, China; gaoyun@xauat.edu.cn (Y.G.); mjy411212@163.com (J.M.); 2 CSCEC Northwest Design and Research Institute Co., Ltd., Xi’an 710018, China; 13572112346@163.com; 3 Wuhan Second Ship Design and Research Institute, Wuhan 430205, China

**Keywords:** reduced graphene oxide filter material, eliminating static electricity, performance differences, electric resistance, sterilization

## Abstract

Improving the filtration efficiency of air filter materials is an ongoing research goal. This study conducted in-depth research on a new reduced graphene oxide air filter material, and the differences in its performance and conductivity durability before and after eliminating static electricity were tested and analyzed. The results showed that the filtration efficiency of the reduced graphene oxide air filter material significantly decreased after eliminating static electricity. The maximum decrease in filtration efficiency was observed at a filtration velocity of 0.8 m/s, with PM_10_ > PM_1.0_ > PM_2.5_. In this case, the filtration efficiency decreased by 11.8%, 7.98%, and 7.17%, respectively. The maximum difference in filtration efficiency of 0.29 μm particulates was about 12.7%. Eliminating static electricity slightly increased the resistance (2.5~15.5 Pa). In addition, the new reduced graphene oxide air filter material exhibited good conductivity and stability after continuous testing. This study provides data support for the application of subsequent electrification sterilization, reference values for multi-angle applications, and the development of new composite air filter materials.

## 1. Introduction

Complex air pollution has always been a key issue of concern [1,2,3], as high concentrations of particulate matter, harmful gases, and microorganisms can cause varying degrees of harm to people and even death [2,3]. The relevant literature shows that particles larger than 10 μm remain in the nasal cavity of the human body [1,2]. Particles between 2 and 10 μm will deposit in various parts of the respiratory system, and about 10% of the particles will deposit in the lungs [1,2]. They will cause related diseases, like pharyngitis or tracheitis. Particles of less than 2 μm may enter the bloodstream of the human body, which will cause more serious problems. Therefore, from a comprehensive perspective, particles of different sizes can cause heart disease, lung disease, respiratory diseases, infectious diseases, etc. [2]. Harmful gases may cause lung diseases in patients, such as chronic bronchitis, asthma, chronic obstructive pulmonary disease (COPD), and respiratory tract infections [2]. The large-scale spread of microorganisms and viruses has brought unprecedented catastrophic effects on the lives and health of people all over the world [3], such as COVID-19 (Corona Virus Disease 2019), and has caused numerous deaths. In addition, toxic and harmful gases, viruses, and bacteria attach to the surface of particulate matter, which allows them to enter the human body and cause a chain reaction whose effects can be life-threatening. With the continuous improvement of people’s living standards and the increasing demand for indoor environmental quality, it is particularly important to create a good and healthy indoor environment.

People are constantly trying various methods and developing new equipment to achieve this goal in response to the above demand. Air filters are widely used because they not only provide clean indoor air after treating outdoor harmful pollutants [4], but are also commonly used to filter the intake air in the internal combustion engines of motor vehicles, which are exposed to polluted air [5]. The main usage areas are concentrated in civil buildings and industrial sites. Civil buildings refer to the places where people live their daily lives, and they are generally used in places with a large number of people, such as supermarkets, schools, airports, offices, hospitals, and so on. Industrial sites often refer to production sites with relatively high environmental requirements, and they are generally suitable for dust prevention in clean workshops, such as workshops, laboratories, and cleanrooms, or for electronic, mechanical, and communication equipment.

At present, many studies have been conducted on air filters [6,7,8,9,10,11,12], and some results have been achieved. The main focus has been on filtration efficiency [6], the use of air filters [7], the effect of combinations of air filter materials [8], the development of new air filter materials [9,10], and so on. In addition, extensive research has also been conducted on the performance of car air intake filter materials [11,12]. However, with the continuous changes in the outdoor environment, adjustments in relevant heating measures, changes in urban structure, personnel migration, and the comprehensive impact of the post-pandemic era, people are paying more attention to composite air filter materials that have high efficiency, low resistance, and bactericidal functions [13]. Research on new air filter materials has always been a hot topic. However, due to the relatively long research cycle, complex preparation processes, high costs, high difficulty in operation, and unstable performance of new air filter materials [14,15], it is difficult to promote new air filter materials on a large scale in a short period of time. In particular, in response to the demand for more frequent use of filters in the post-pandemic era [16], the performance requirements for air filters will become higher compared to the past.

Ordinary air fiber filter materials mainly rely on mechanical blocking effects such as diffusion effect, interception effect, and inertia effect to filter particles in the air. As a result, the filtration efficiency is not significant for small particle sizes. For most bacteria and viral microorganisms, the size is on the micron or submicron scale. For example, relevant studies have shown that viruses are about 100 nm in size [16]. These small particles in the air could be efficiently captured by the filter fibers, which are very small and dense. However, it will also increase the air resistance of the filter materials, which will increase the operating energy consumption and cost of the air filter. Electret fiber materials typically carry voltages ranging from hundreds to thousands of volts and have very small fiber gaps, which will result in the formation of numerous passive electrodes. The electric field between the electrodes can reach tens of 0–50 MV/m or even higher [17]. Therefore, in addition to their original mechanical blocking effect, electret air filter materials can also rely on electrostatic force to directly attract charged particles in the air and capture them. Maybe also induce neutral particles in the air to generate polarity and then capture them, thereby more effectively filtration of submicron particles in the air and significantly improving filtration efficiency without increasing air resistance.

Therefore, electret materials [17,18] are currently one of the main filtration materials used in the market due to their unique characteristics. However, there is still a problem that the static electricity gradually decreases or even disappears after a period of use, until only the mechanical filtration is left [19]. It is more important and of practical application value to develop new air filter materials with electrostatic effects. At present, porous media are often used as raw materials to synthesize new materials [20,21,22,23], such as carbon black [20], graphene [21], graphene oxide [22,23], and carbon nanotube materials [24]. Although extensive preparation and research have been conducted on new materials, there is currently a lack of research on their electrostatic properties and the differences in their performance before and after eliminating static electricity. Graphene and its derivatives have become a hot topic in the synthesis of materials due to their unique characteristics [22,23]. They are widely used in the fields of mobile devices, aerospace, environmental protection, and new energy batteries, such as manufacturing medical disinfectants and food packaging, new super strong materials, making transparent touch screens, transparent panels, ultra light aircraft materials, ultra tough bulletproof vests, and air filter materials. However, there are currently few studies on the performance of reduced graphene oxide air filter materials before and after eliminating static electricity, with more emphasis on improving the effectiveness of filtering particulate matter. In addition, relevant studies have found that the structure of reduced graphene oxide is more relatively stable [25], which makes it difficult to combine it with other substances and induces conductivity and thermal conductivity properties. Therefore, it is not known whether new reduced graphene oxide air filter materials can be electrified to achieve the same filtration effect as an electret material, whether they can effectively solve the problem of loss of static electricity, or whether they can heat up the fiber surface after electrification to damage the cell structure of bacteria and other microorganisms and achieve a sterilization effect. As a result, there is not enough research on the differences in the performance and conductivity durability of reduced graphene oxide filter materials before and after eliminating static electricity.

Therefore, this study focused on the above practical issues and conducted in-depth research on the differences in the performance and conductivity durability of a new reduced graphene oxide filter material before and after eliminating static electricity. It provides reference values for multi-angle applications and the development of new composite filter materials.

## 2. Methods

### 2.1. Material Selection

Two clean, reduced graphene oxide air filter materials were selected as experimental materials in order to make the experimental purpose clearer and the experimental results more accurate; pieces with dimensions of 5 cm × 5 cm were tested. One piece was the blank group, where the static electricity was eliminated from the reduced graphene oxide filter material. Another piece was completely immersed in isopropanol for 2 min to remove the static electricity on the surface of the fiber [26]. Next, it was ventilated and dried in a cool place for 24 h without touching any surface, and it is referred to as the material after eliminating static electricity.

### 2.2. Experimental Systems

**Figure 1:**
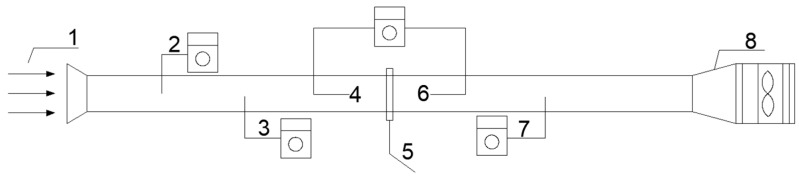
Experimental setup. 1, inlet airflow; 2, flow velocity measurement point; 3, pressure measurement point at the front of the filter material; 4, particle measurement point at the front of the filter material; 5, filter material; 6, particle measurement point at the end of the filter material; 7, pressure measurement point at the end of the filter material; 8, fan.

An experimental setup was built according to the experimental requirements [14], as shown in Figure 1. A GRIMM1.109 Portable Aerosol Spectrometer was used to measure the concentration of particles before and after the air filters were applied, and it was supplied by Beijing Saak-Mar Environmental Instrument Ltd., Beijing, China. The upper limit for the concentration measurement was 2,000,000 P/L, the measurement range was 0.1~100,000 μg/m^3^, and the repeatability was 5%. An HD2114P.0 Portable Micromanometer was used to measure filtration resistance, and it was supplied by DeltaOHM Co., Ltd., Padua, Italy, with an accuracy of ±2% reading +0.1 m/s. The pressure range was ±0.4% F.S. An HD37AB1347 Indoor Air Quality Monitor was used to measure velocity, and it was supplied by DeltaOHM Co., Ltd., Padua, Italy, with an accuracy range of ±3%. A JSM-6510LV scanning electron microscope was used for analysis, and it was supplied by Japan Electronics Co., Ltd., Tokyo, Japan, its magnification was 5~30 million times, and its resolution was up to 3.0 nm. A TSI7525 Indoor Air Quality Meter Measuring Instrument was used to measure temperature and humidity, and it was supplied by TSI Instrument Beijing Co., Ltd., Beijing, China. The temperature measurement range was 0~60 °C, with a measurement accuracy of ±0.6 °C and 0.1 °C resolution. The relative humidity measurement range was 5~95% RH, measurement accuracy was ±3% RH, and the resolution was 0.1% RH. The average concentrations over 5 min before and after the testing were used in the calculations to reduce experimental errors [27].

### 2.3. Performance Parameters

The air filters’ filtration efficiency was calculated using Equation (1) [14].

(1) $$\eta =\frac{C_{1}-C_{2}}{C_{1}}\times 100\%$$

where η is the filtration efficiency (%); *C*_1_ is the concentration of particulate matter before filtration (μg/m^3^); and *C*_2_ is the concentration of particulate matter after filtration (μg/m^3^).

The filtration velocity before and after the filters were applied were the same, and the cross-sectional area was equal. The filtration resistance could be expressed by the static pressure difference. The filtration resistance was calculated using Equation (2) [14].

(2) $$\Delta P=P_{2}-P_{1}$$

where *P*_1_ is the static pressure before filtration (Pa) and *P*_2_ is the static pressure after filtration (Pa).

## 3. Results and Discussion

### 3.1. Distribution of Atmospheric Particle Concentrations

**Figure 2:**
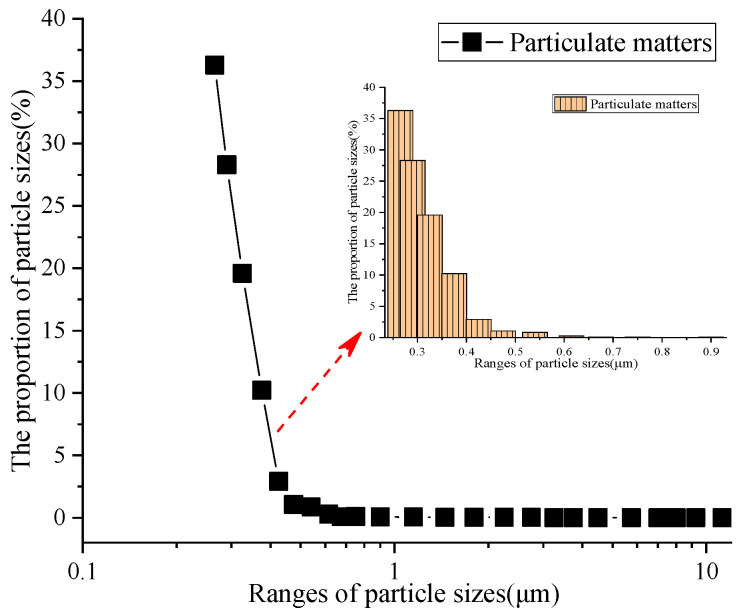
Particle size distribution of atmospheric air.

The particle size distributions using outdoor atmospheric air as the dust source in this paper are shown in Figure 2, which makes the experimental results more consistent with the actual effect.

As shown in Figure 2, there are significant differences in the proportion of particulate matter under different particle sizes, among which 0~1.0 μm particles accounted for the majority of the particles, about 99.78%. Particles with sizes greater than 1.0 μm accounted for approximately 0.218%, which is consistent with the results in ref. [28]. Furthermore, it was found that particles with sizes less than 0.5 μm accounted for about 98.4%. The atmosphere of Xi’an during the testing period was mainly composed of small-sized particles [29]; these particles were more likely to enter the human body, which might lead to varying degrees of health problems and even death [2]. Therefore, there is an urgent need to improve the purification efficiency of filters for small-sized particles.

### 3.2. The Influence of Filtration Velocity

The filtration velocity range of 0.2~1.0 m/s was selected according to China’s national standard [29]. The changes in filtration efficiency of the new reduced graphene oxide air filter material under different filtration velocities before and after eliminating static electricity are shown in Table 1.

Table 1 shows that with the increase in filtration velocity, the new reduced graphene oxide air filter material before and after eliminating static electricity showed a trend of first increasing and then decreasing. The main reason is that the range of filtration velocity during the experimental process is in the joint action area of the interception effect and the inertia effect [14]. As the filtration velocity increases, the inertia effect increases while also increasing the interception effect. At this point, the diffusion effect decreases accordingly. As a result, it increases the capture efficiency of particulate matter when the filtration velocity increases to a certain value and the disturbance of the airflow between fibers increases the inertia force of certain particles. It may make the interception effect further weaken. The strong airflow disturbance may also cause the intercepted particles to fall off, resulting in a decrease in capture efficiency.

**Figure 3:**
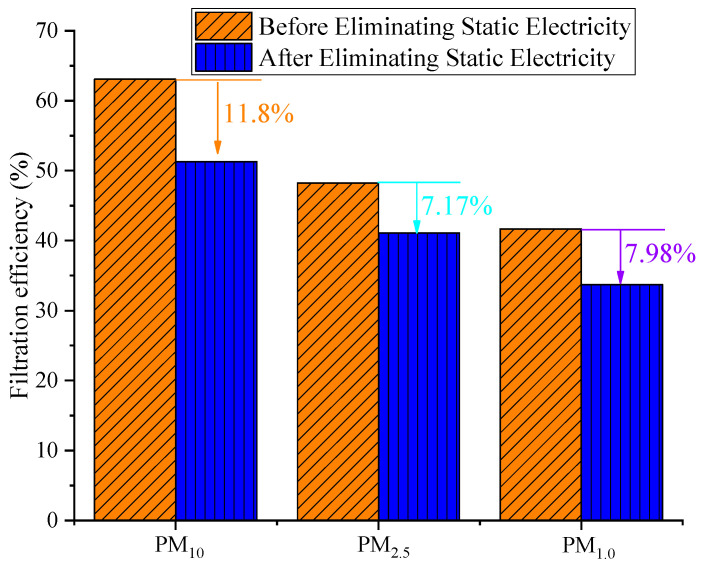
Filtration efficiency of reduced graphene oxide air filter material before and after eliminating static electricity.

The filtration efficiency range of the reduced graphene oxide air filter material for PM_10_ before eliminating static electricity was 55.3% to 63.1%, the filtration range for PM_2.5_ was 40.0% to 48.3%, and the filtration range for PM_1.0_ was 31.5% to 41.7%. Meanwhile, after eliminating static electricity, the filtration efficiency range for PM_10_ was 46.8% to 51.3%, the filtration range for PM_2.5_ was 35.7% to 41.1%, and the filtration range for PM_1.0_ was 27.9% to 33.7%. Thus, the filtration efficiency of the reduced graphene oxide air filter material after eliminating static electricity was significantly decreased compared to that before eliminating static electricity. The filtration efficiency for PM_10_ decreased by 8.5% to 11.8%, the filtration efficiency for PM_2.5_ decreased by 4.3% to 7.2%, and the filtration efficiency for PM_1.0_ decreased by 3.6% to 8.0%, with the greatest impact on PM_10_. This is because the isopropanol solution neutralizes the positive and negative charges on the fibers, causing their charges to permanently decay [30]. After eliminating static electricity, the surface of the fiber does not carry static electricity, causing the fiber to become inactive. As a result, the particle capture efficiency is significantly lower than before eliminating static electricity. Similar experimental results have been published [31], verifying our results. In addition, it can be seen that the filtration efficiency of the different materials reached its maximum value at 0.8 m/s. The filtration efficiency of the new reduced graphene oxide filter material for PM_10_, PM_2.5_, and PM_1.0_ before eliminating static electricity was 63.1%, 48.3%, and 41.7%, respectively. After eliminating static electricity, the filtration efficiency of the new reduced graphene oxide filter material for PM_10_, PM_2.5_, and PM_1.0_ was 51.3%, 41.1%, and 33.7%, respectively. Figure 3 shows the difference in performance of the reduced graphene oxide air filter material before and after eliminating static electricity at the optimal filtration velocity.

From Figure 3, it can be seen that the filtration efficiency of the reduced graphene oxide air filter material after eliminating static electricity significantly decreased, with the degree of decrease in the order of PM_10_ > PM_1.0_ > PM_2.5_ (11.8%, 7.98%, and 7.17%, respectively). The main reason for this is that, after eliminating static electricity, the charges carried on the fibers are neutralized, resulting in a decrease in surface charge. This indicates that the filtration of fine particles mainly relies on electrostatic effects, while the filtration of large particles relies on other filtration mechanisms such as inertial collision. This conclusion is consistent with the literature [32] and indirectly indicates that adding static electricity to the filter material is beneficial for improving the capture of particulate matter, thereby creating a good indoor environment. At the same time, it also provides a reference for the extension of the same type of materials or the addition of electrostatic function to the new multifunctional composite materials in the future.

### 3.3. Differences in Counting Filtration Efficiency for Different Particle Sizes

**Figure 4:**
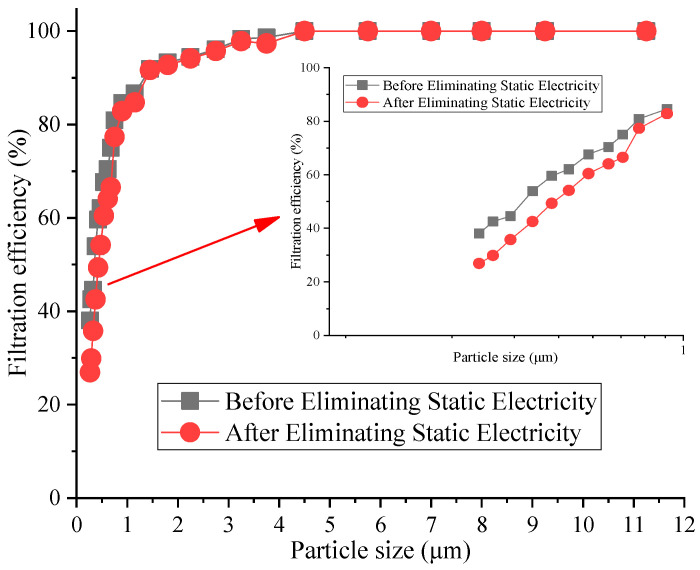
Filtration efficiency of reduced graphene oxide filter material by particle size.

The counting filtration efficiency of the reduced graphene oxide air filter material before and after eliminating static electricity at the optimal filtration velocity is shown in Figure 4.

Figure 4 shows that the filtration efficiency of the reduced graphene oxide air filter material decreased significantly after eliminating static electricity. The filtration efficiency of 0~1.0 μm particles was the highest, ranging from 1.75% to 12.7%, while the difference for large particles was not significant. The maximum difference in filtration efficiency was for 0.29 μm particulates, at about 12.7%. These results show that the filtration efficiency of small particles mainly depends on electrostatic effects. The relevant literature shows that electrostatic discharge or washing can significantly reduce the filtration characteristics of the air filter material [33]. This is because the electrostatic effect of the reduced graphene oxide air filter material disappears after eliminating static electricity, and only the mechanical filtration is left, resulting in a decrease in filtration efficiency.

### 3.4. The Change in Filtration Resistance with Filtration Velocity

**Figure 5:**
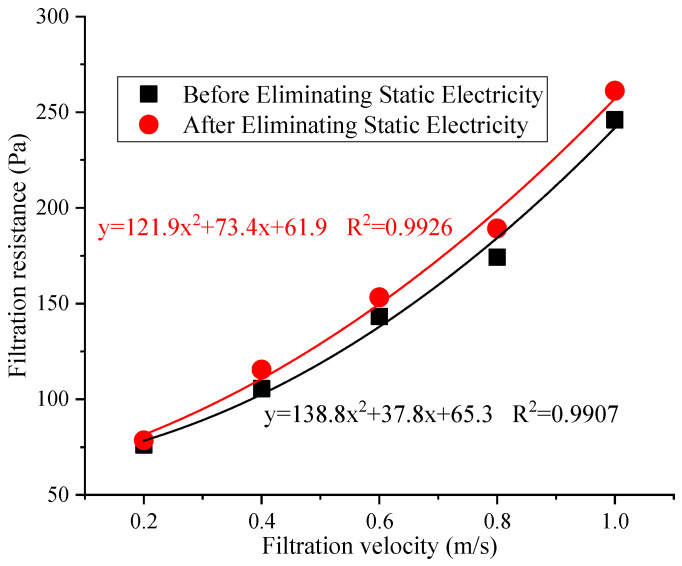
Changes in filtration resistance of reduced graphene oxide air filter material before and after eliminating static electricity.

The difference in filtration resistance of the reduced graphene oxide air filter material before and after eliminating static electricity is shown in Figure 5.

From the figure, it can be seen that the filtration resistance of the reduced graphene oxide air filter material increased with the increase in filtration velocity before and after eliminating static electricity. Within the tested filtration velocity range, the resistance range before eliminating static electricity was 76~246 Pa, while the resistance range after eliminating static electricity was 78.5–261.5 Pa. Thus, the resistance of the reduced graphene oxide air filter material after eliminating static electricity was slightly higher than that before eliminating static electricity (a difference of 2.5~15.5 Pa). The main reason is that soaking the reduced graphene oxide air filter material in an isopropanol solution has a certain impact on the internal micropores. As a result, it causes a certain degree of contraction and binding between fibers, affecting the uniformity of the airflow velocity. The resistance cannot effectively pass through the fibers after colliding under the same air flow, resulting in an increase in its resistance, which is consistent with the conclusions in the literature [23]. Therefore, it is necessary to consider both resistance and filtration efficiency in practical applications.

### 3.5. Changes in the Electric Resistance of Reduced Graphene Oxide Air Filter Material

**Figure 6:**
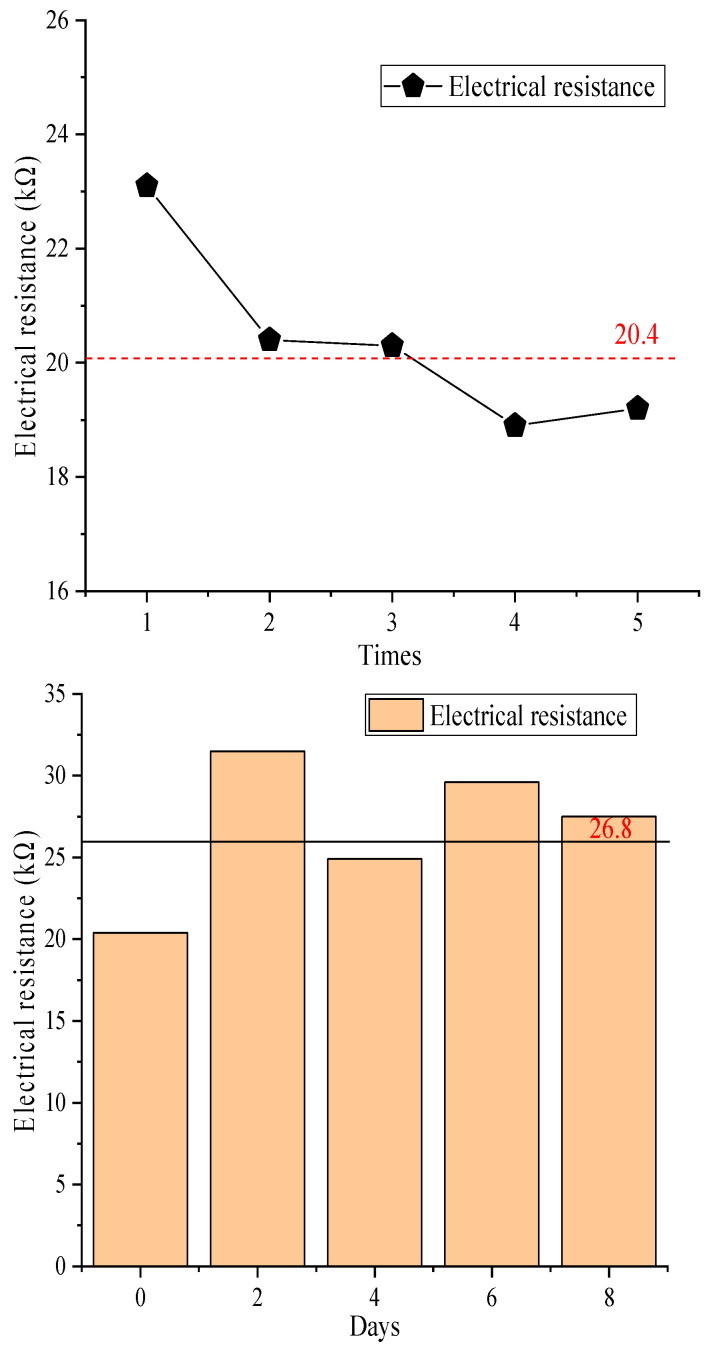
Electric resistance of reduced graphene oxide filter material.

The electric resistance characteristics of the reduced graphene oxide air filter material and its conductivity need to be investigated further. The fiber electric resistance values of the reduced graphene oxide air filter material and the variation in fiber electric resistance values over time are shown in Figure 6.

**Figure 7:**
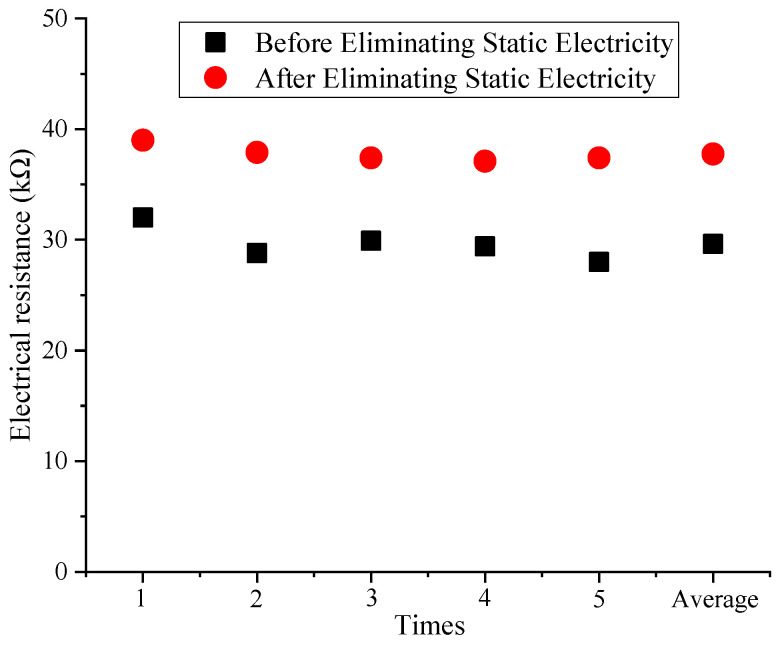
Electric resistance values before and after eliminating static electricity.

Figure 6 shows that the electric resistance value of the reduced graphene oxide air filter material exhibited certain fluctuations after different test durations, which ranged from 18.9 KΩ to 23.1 KΩ, with an average of 20.4 KΩ. These good conductivity characteristics are because reduced graphene oxide is filled between the fibers, completely enveloping them [34], and the electric resistance changes were not significant under the same conditions. Therefore, the reduced graphene oxide air filter material has good conductivity and continuous stability, which also indirectly confirms that adding charges can improve the pollutant capture efficiency of existing composite materials [35]. As the number of days increased, the material still exhibited relatively low electric resistance values and good conductivity, which provides a foundation for the subsequent capture of small particles and expands the application of new reduced graphene oxide air filter materials. The fiber electric resistance of the new reduced graphene oxide air filter material before and after eliminating static electricity is shown in Figure 7.

**Figure 8:**
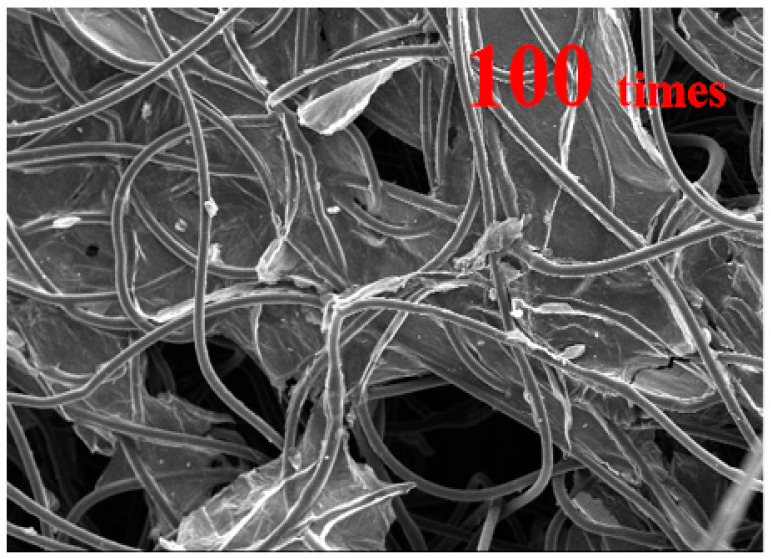
Scanning electron microscope image of reduced graphite oxide air filter material.

From Figure 7, it can be seen that the electric resistance value after eliminating static electricity was greater than the electric resistance value before eliminating static electricity, with an overall increase of 7.0 KΩ to 9.4 KΩ and an average of 8.1 KΩ. This is because the process of eliminating static electricity has a certain impact on the fiber structure [30], leading to damage to the reduced graphene oxide coating and part of the fiber structure breaking, which makes it difficult to form a complete circuit between the fibers. As a result, based on the above actual situation, the overall increase in fiber surface resistance has been caused. To illustrate the fiber structure of the new reduced graphene oxide air filter material, a scanning electron microscope image is shown in Figure 8.

From the figure, it can be seen that an enveloping layer of reduced graphene oxide covered the surface of the reduced graphene oxide air filter material fibers, and some sections of reduced graphite oxide were cross-linked between the surfaces. This can provide a foundation for achieving fiber surface conductivity [34]. In addition, the relevant literature also found the use of fiber electrification to achieve a sterilization function while also effectively solving the problem of electret materials losing their static electricity after a period of use [36]. Specifically, during the process of fiber electrification, continuous electrification generates heat on the surface of the fiber, which heats up the fiber surface. When a certain temperature is reached, it causes some bacteria and viruses attached to the fiber surface to reach the death temperature, which causes the cellular structure to lose water within the cell at its relative high temperature. As a result, the microorganisms have been killed in this way. But it is also necessary to consider both the duration of power on and the temperature of the fiber surface to avoid damaging the structure of the fiber itself, which results in its overall effect not meeting practical needs. The effect of conductivity is also related to the concentration of graphene oxide used to prepare the new reduced graphene oxide air filter material [37]. The high concentration can improve conductivity, but it can also cause fiber to harden and increase filtration resistance. While the concentration is too low, it will result in weak conductivity and an inability to achieve fiber conductivity. At the same time, it will also cause a weak reduction effect of reduced graphene oxide coating and detachment phenomenon. Therefore, it is needed for in-depth research on the concentration ratio. In summary, composite new materials will be widely used in various site environments, such as underground mining environments, and so on [38,39].

## 4. Conclusions

The difference in the performance and conductivity durability of the new reduced graphene oxide air filter material before and after eliminating static electricity were measured and analyzed in this paper. The conclusions are as follows:

1. The filtration efficiency of the reduced graphene oxide air filter material after eliminating static electricity significantly decreased compared to before eliminating static electricity. The filtration efficiency for PM_10_ decreased by 8.5% to 11.8%, the filtration efficiency for PM_2.5_ decreased by 4.3% to 7.2%, and the filtration efficiency for PM_1.0_ decreased by 3.6% to 8.0%, with the greatest impact for PM_10_. The degree of decrease was in the order of PM_10_ > PM_1.0_ > PM_2.5_ (decreased by 11.8%, 7.98%, and 7.17%, respectively).
2. The counting filtration efficiency of the new reduced graphene oxide air filter material decreased significantly after eliminating static electricity. The filtration efficiency of 0~1.0 μm particles was the highest, with the efficiency ranging from 1.75% to 12.7%, while the difference was not significant for large particles. The maximum difference in filtration efficiency was for 0.29 μm particulates, at about 12.7%.
3. The resistance after eliminating static electricity was slightly higher than that before (an increase of 2.5~15.5 Pa). As the number of days increased, it still exhibited relatively low electric resistance values and good conductivity.
4. The electric resistance value after eliminating static electricity was greater than that before eliminating static electricity, with an overall increase of 7.0 KΩ to 9.4 KΩ and an average of 8.1 KΩ.

## Figures and Tables

**Table 1:** Filtration efficiency under different filtration velocities.

Content	Particulate Matter	Filtration Velocity (m/s)				
		0.2	0.4	0.6	0.8	1.0
Before Eliminating Static Electricity	PM_10_	55.3	57.7	61.2	63.1	58.4
	PM_2.5_	40.0	42.6	44.6	48.3	42.3
	PM_1.0_	31.5	32.9	37.1	41.7	36.2
After Eliminating Static Electricity	PM_10_	46.8	47.9	49.8	51.3	47.4
	PM_2.5_	35.7	37.7	38.7	41.1	36.3
	PM_1.0_	27.9	29.0	32.9	33.7	28.8

Note: Standard Atmosphere (ISA) values at SL pressure *p* = 1.013250 × 10^5^ Pa (760 mm Hg). The average temperature was 22.6~33.7 °C, and the average humidity was 32.8~51.9%.

## Data Availability

No new data were created or analyzed in this study. Data sharing is not applicable to this article.
